# Dynamic consent: a patient interface for twenty-first century research networks

**DOI:** 10.1038/ejhg.2014.71

**Published:** 2014-05-07

**Authors:** Jane Kaye, Edgar A Whitley, David Lund, Michael Morrison, Harriet Teare, Karen Melham

**Affiliations:** 1 Centre for Health, Law and Emerging Technologies (HeLEX), Nuffield Department of Population Health, University of Oxford, Oxford, UK; 2 Information Systems and Innovation Group, Department of Management, London School of Economics and Political Science, London, UK; 3 HW Communications Ltd, Parkfield, Lancaster, UK

## Abstract

Biomedical research is being transformed through the application of information technologies that allow ever greater amounts of data to be shared on an unprecedented scale. However, the methods for involving participants have not kept pace with changes in research capability. In an era when information is shared digitally at the global level, mechanisms of informed consent remain static, paper-based and organised around national boundaries and legal frameworks. Dynamic consent (DC) is both a specific project and a wider concept that offers a new approach to consent; one designed to meet the needs of the twenty-first century research landscape. At the heart of DC is a personalised, digital communication interface that connects researchers and participants, placing participants at the heart of decision making. The interface facilitates two-way communication to stimulate a more engaged, informed and scientifically literate participant population where individuals can tailor and manage their own consent preferences. The technical architecture of DC includes components that can securely encrypt sensitive data and allow participant consent preferences to travel with their data and samples when they are shared with third parties. In addition to improving transparency and public trust, this system benefits researchers by streamlining recruitment and enabling more efficient participant recontact. DC has mainly been developed in biobanking contexts, but it also has potential application in other domains for a variety of purposes.

## INTRODUCTION

The twenty-first century has been marked by considerable innovation in the field of information technology (IT), which has led to rapid changes in the way that biomedical research is carried out.^1^ Biomedical research is now heavily dependent upon infrastructures such as biobanks and data repositories and is ‘increasingly of a global nature with data and samples exchanged, accumulated and created through a number of dynamic research networks and consortia that involve multi-disciplinary teams located in different countries'.^2^ This new way of doing research has been accompanied by the development of new ethical norms, practices and standards particularly in the area of consent.^3^ Despite innovations in the use of technology to develop research capabilities, the potential to use technology to address some of the ethical and legal issues around research participation has not been fully realised.^4^

This paper describes the dynamic consent model, which is an example of how IT can be used to satisfy the legal and regulatory requirements for research consent, while at the same time providing a personalised communication interface for interacting with patients, participants and citizens. It was initially developed in the field of biobanking but has the potential to be applied more broadly to situations where there are multiple and varied uses of data requiring different kinds of consent over a long period of time. Therefore, ‘dynamic consent' refers to a specific project in the field of biobanking but is also a more general concept and approach that has the potential to radically change the nature of participation in both clinical care and research.

## THE CHALLENGE OF CONSENT

The requirement that researchers obtain informed consent from potential participants before research commences is a fundamental principle of medical research, enshrined in the Declaration of Helsinki and subsequent legal instruments.^5, 6^ The requirement for consent is underpinned by ethical principles of respect for persons and individual autonomy. Consent is also the basis for data protection and privacy law in most countries although there may be exceptions for medical research.^7^

The consent form has become the primary means of recording individual involvement in research, and for determining ‘whether additional consent is required, or whether the existing consent covers the new research'.^8^ As such, it formalises part of the implicit social contract between the public and researchers. New forms of biomedical research and the ease by which multiple data from diverse sources can now be collected, stored, analysed and shared in greater volumes than ever before^9, 10, 11^ challenge the meaning of informed consent and call into question whether the existing processes of engaging with participants are still appropriate for these new dynamic ways of undertaking research.

In the case of biobanks where there are multiple researchers and research projects, it is difficult to obtain informed consent for all future research uses at the time of recruitment into the biobank or before such research commences, as is required in the original formulations of the Declaration of Helsinki.^12^ Re-consenting is costly and time-consuming, and difficulty in locating people can result in high drop-out rates. As a practical solution, broad consent is often obtained with participants agreeing to the ‘rules of the game' that the material may be shared widely and used by many researchers.^3, 13, 14^ There are a number of reasons, however, why this approach is inadequate to meet the demands of meaningfully informed consent. Unlike traditional research, there is no single ‘experimental procedure' with fixed duration, end points or outputs, in which individuals are being asked to participate. Instead, individuals are being asked to consent to involvement in ongoing research infrastructures with multiple, emergent research questions and methods, where the potential privacy risks of personal and individually identifying health information may be a greater concern than the risk of physical harm.^15, 16, 17^ As the nature of biomedical research changes, the social contract between participants and researchers needs to evolve with it. If biobank research is open-ended and ongoing then information technologies offer the possibility for participant involvement similarly to extend through time. Individuals need no longer be passive human ‘subjects' but can be engaged over time and recognised as active, interested and valued research participants.^4, 18, 19^

The expression of individual autonomy is also not static: it involves making choices and decisions, such as the decision to consent to participate in research, over the course of one's lifetime. Indeed, current guidelines already recognise consent as a process; an ongoing interaction between researcher and participant.^20, 21^ The paper-based tools that have been used to record consent have, however, limited ongoing engagement.^22^ ‘Respect for persons' means giving individuals as much choice and control in what is done with their personal information and material as is reasonably achievable.^23^ This principle is reflected in case law; where the courts have found that ‘subject to [certain] qualifications … an individual's personal autonomy makes him – should make him – master of all those facts about his own identity, such as his name, health, sexuality, ethnicity, his own image … and also of the ‘zone of interaction' … between himself and others'.^24^ This principle is also evident in the proposed European Data Protection Regulations.^25^

Many longitudinal biobanks, which inherently involve an ongoing engagement with participants, have already recognised the limitations of a one-off static consent and employ ongoing monitoring and governance mechanisms to enable participant recontact over time. These mechanisms represent transitional movements along the continuum from broad to tiered or more flexible forms of consent. Examples of best practice, such as UK Biobank and the Norwegian Mother Child Cohort Study, rely on a broad consent but also provide regular updates to participants on ongoing biobanking activity.^26^ However, for many biobanks these services are seen as patient communication ‘extras', and there is little attention to the possibilities of information technologies to enable dialogue and engagement between researchers and participants.

Technological advances are also enabling the collection of richer and more comprehensive ‘personalised' data sets. Many of the traditional protections used in research such as anonymisation, coding and pseudonymisation are increasingly tested or rendered ineffectual by advanced data collection.^17, 27, 28^ The potential that individuals can be identified either directly or indirectly from the data may invoke the requirement for explicit consent under data protection and privacy law.^29^ The current proposals for the European Data Protection Regulations suggest the requirement for medical research will be explicit consent,^25^ which brings into question whether broad consent will remain a lawful option for research.

As informatics technology becomes more powerful and the ability to link different data sets increases, the static, paper-based systems for recording consent are no longer fit for purpose. New approaches are needed to meet ethical and legal requirements for consent and to accommodate the fluidity of data flows in research networks. Dynamic consent addresses the changing nature of biomedical research and allows researchers to overcome the limitations of static consent, the increasing obsolescence of anonymity and the fluctuating requirements of the legal and regulatory environment.

## WHAT IS DYNAMIC CONSENT?

[Boxed-text bx1] Dynamic consent is a personalised, communication interface to enable greater participant engagement in clinical and research activities. It is a participant-centred initiative that places patients and research participants at the centre of decision making, providing an interactive IT interface to engage with participants.^4^ This approach is ‘dynamic' because it allows interactions over time; it enables participants to consent to new projects or to alter their consent choices in real time as their circumstances change and to have confidence that these changed choices will take effect.

Rather than being restricted to the opportunity only to give broad consent to the use of their samples and data, individuals could provide different types of consent depending upon the kind of study. These consent preferences travel securely with their samples or data so that third parties know the scope of the consent that applies. A secure consent interface allows participants to change their consent preferences reliably. It also allows them to alter their contact details, receive information on the use of their samples and data, enrol in new studies and complete online surveys. This allows them to engage with the research study in their own time, as much or as little as they choose. Available preferences can be adapted to suit the capabilities and needs of institutions, researchers and participants.[Boxed-text bx2]

### Different consents

By using a technology-based platform, the consent process is not, as is often the case with paper-based documentation of consent, locked in time to the beginning of the research process. Depending upon the nature of the research enterprise, participants could consent to a broad range of uses of their samples and data, or opt to be approached on a case-by-case basis, or set different preferences for different types of research. These preferences can be ‘opt ins' or ‘opt outs': participants can tailor their profiles to receive no information for specified periods of time or to give a broad consent if they so wish. The options offered to participants can be set by the biobank or researcher according to their requirements.

Reliable storage and enforcement of consent choices is achieved by electronically and cryptographically ‘wrapping' the individual's consent preferences with samples/information provided. This is possible because of machine-readable disclosure policies or ‘sticky policies' that attach to data.^30^ This package of ‘wrapped information', which contains specific consent provisions, travels with a participant or donor's data and samples as these are shared or accessed for different purposes. ‘Wrapped Information' embraces new homomorphic encryption techniques,^31^ which allow information to be processed in its encrypted state, while permitting the results of the processing to remain encrypted. At a later stage, the encrypted result of the processing can be decrypted to disclose the result. In this case, the processing itself never decrypts identifiable information but is still able to generate analytical results from the encrypted data. Homomorphic encryption provides new, privacy-enhancing, technical capabilities allowing the processing of sensitive information through the dynamic consent interface.

It has been suggested that dynamic consent will necessitate participants giving consent ‘over and over again' to each new development in a biobank, whether serious or trivial, and that this fails to respect the choice of those who prefer to participate passively and may lead to a ‘consent fatigue' where participants become disengaged and their consent rendered less meaningful.^26^ However, this presents a misleading view of dynamic consent as positioned in opposition to broad consent. Dynamic consent is not a replacement for existing models such as broad consent but rather a facilitation tool to improve how that consent is obtained, understood and acted upon. The ‘opt in' and ‘opt out' approach to choice means that a participant can still choose to give a broad consent and not receive updates and so on, but if at some future point they wish to become more engaged they have the option to do so. Similarly, if a participant finds that they wish to take a break from interacting with a biobank, perhaps in response to a worsening of a chronic condition, the dynamic consent interface allows this option.

### Tailored

The dynamic consent interface acts as a personalised communication interface, a source of information and a platform for modifying consent preferences.^32^ All aspects of the interface can be tailored to individuals' needs. Participants can select how they prefer to be contacted and kept informed, from traditional paper-based formats such as letters or newsletters to email, SMS (text message) alerts or via social networking sites, depending upon the capabilities and resources of the biobank. They can select how often they wish to be contacted and what types of information they are primarily interested in receiving. For example, individuals may opt to receive updates on what their biobank is doing, what new studies are seeking participants or other kinds of feedback and information. Using a technology platform also allows individuals to keep track of all of their activities within one accessible interface. This can be accessed at any time, outside of the clinical or research setting and without the burden of storing and having to navigate a potentially substantial archive of paper documents.

A recurring problem for most, if not all, IT-based innovation in the biomedical context is the so-called ‘digital divide'.^33^ Access to the internet is not equally distributed throughout (or between) populations and is influenced by a range of factors, including age, socio-economic status and health. Online services run the risk of excluding individuals and communities with limited or no internet access. Although dynamic consent cannot claim to provide a solution to all the problems of the digital divide, there are ways in which it can be tailored to increase inclusiveness. The dynamic consent interface can run on tablet computers, websites and also has the potential to be configured for mobile phones in order to address differing usage needs and preferences of participants. Mobile or ‘m-health' options have significant potential to reach vulnerable or elderly adults and facilitate access to health information in developing countries where mobile coverage is far more widespread than other forms of digital infrastructure.^34^ Further, a dynamic consent interface can be installed on a device that is delegated for use by a carer or family member or that can be utilised in the clinic with a health professional on hand to provide assistance.^35^ It also should be run alongside more traditional face-to-face interactions with patients.

### Customised to research needs

Dynamic consent explicitly incorporates a flexible, configurable design accommodating both participant and researcher needs. Biobanks, cohorts, clinical trials and consortia have different interactions with individuals and need to be able to tailor the available consent, information and communication settings accordingly. Each instantiation of the system can be tailored to meet the needs of each particular research enterprise, ensuring that settings are appropriate and fit for purpose to suit both the research and the study population.

This flexibility also extends to the interaction with existing biobank infrastructures; dynamic consent technology can be configured to work with a wide range of Laboratory Information Management Systems and other data storage architectures. Biobanks will not need to replace existing data management and storage infrastructure to implement a dynamic consent system. There will, of course, be costs associated with implementing dynamic consent. These include financial costs of acquiring and installing the technology as well as work associated with understanding, specifying and implementing specific consent options for research settings. It takes time and effort for biobank staff and participants to learn how to use the interface, and the interface needs to be populated with relevant and appropriately tailored content, for example, links to podcasts and online information resources. However, these costs can be offset in the long term as dynamic consent is not simply a system for improving consent; it also tracks and stores participant information (including preferences), facilitates engagement and provides a range of additional benefits as set out in subsequent sections of this paper. As the practical implementation of dynamic consent systems progresses, more information will become available on the specific costs and optimal approaches for installation, which will enable detailed cost-benefit analyses.[Boxed-text bx3]

## BENEFITS OF DYNAMIC CONSENT

By understanding and supporting biomedical research as a partnership between participants and researchers, dynamic consent makes possible better research and a better research experience for both parties. The interface offers participants a responsive format through which to become involved with research; one which respects their autonomy by enabling information and consent preferences to be exercised and ultimately providing more meaningful consent. This in turn benefits researchers by facilitating more engaged participant populations, streamlining recruitment and improving public trust.^36^ Easy configurability allows future possibilities for integrating research and clinical care, facilitating translational research and improving health-care outcomes and reducing the costs of research in the long term.

### Streamlines recruitment

The costs of recruitment into research are high, and recruitment rates into publicly funded studies are relatively low. Patients are, however, very willing to participate in research. In a recent study of 203 adult patients, 69% wanted to take part in biobank studies.^37^ The primary benefit of the dynamic consent model for researchers is that it simplifies and streamlines consent and recruitment processes. For each new research request, the dynamic consent software can automatically select participants who are willing to be involved. Its interactive functionality provides an easy mechanism for individuals to be identified, approached and recruited for new studies, to participate in online surveys or to canvas opinions on a range of concerns. This makes recruitment less costly, reducing paperwork and staff time. People are willing to be engaged in research, but current recruitment processes are not effective. An on-going interaction and communication capability has the potential to address this imbalance.

### Enables efficient recontact

Maintaining contact with participants helps researchers to deal with many of the ethical and legal problems that emerge from unforeseen circumstances. Currently, it is often impractical to recontact individuals to obtain new or updated consent when circumstances change. If a judgement is made that the new research is outside of the original consent and re-consenting is impractical, research ethics committees and national regulatory bodies are often asked to represent participant interests. Dynamic consent makes it easy to contact participants and to provide readily accessible information so that people can make their own informed decision. However, this does not mean that dynamic consent should replace the human contact of original sign-up nor the opportunity for participants to discuss the process of re-consenting face to face with health professionals.

### Conforms to the highest legal standards

Freely given, ‘informed' consent is a unanimous requirement of biomedical law, privacy and information legislation across the world. There are also a number of exemptions from consent for medical research that exist in law, such as public health surveillance. However, the exemptions in law are always subject to change as evidenced by the latest proposals to European Data Protection law.^25, 38^ The increased ease of participant recontact with a dynamic consent model offers a flexible and responsive way to deal with changing legal and ethical requirements. The built-in compliance framework also ensures adherence to the highest standards in data privacy protection and risk assessment, going further than the current standard required by many international legal requirements and privacy impact assessments.

### Fine grained withdrawal

Participants in research have a right to withdraw by requesting that their samples and data not be made available for research and, in some cases, that their samples and/or data be destroyed. However, quite often this withdrawal is ‘all or nothing'. In practice, there is often no option to limit withdrawal to specific research that an individual might find objectionable. The choice is limited to withdrawing from the biobank entirely or giving broad consent for samples and data to be available for all research requests that are subsequently approved by the biobank. Dynamic consent enables choice to be more nuanced and may mean that research participants will be less likely to withdraw from a biobank infrastructure than if they have only an ‘all or nothing' choice.

### Enables better communication

Traditional forms of obtaining consent engage participants at the outset of the study; however, these are rarely the best mechanisms to maintain contact with participants or to convey research findings to those whose material made the research possible. Participants may or may not want access to scientific reports and publications but may wonder whatever became of the project to which they contributed. The dynamic consent interface allows general research results to be returned to participants according to their preferences, either as a simple ‘thank you' for their involvement or by informing them how their samples and information have been used. Through this interface, it is possible for participants to link into a wealth of information that already exists for other purposes, such as lay descriptions required for grant applications and ethics approval. It also provides broader possibilities for engagement than just the information sheet that accompanies the consent form. This can add value when recruiting previously hard-to-reach audiences, including children, service users where English is not their first language or participants with cognitive, visual or audio impairment. Indeed, hyperlinks to various alternative forms of presentation may contribute to overcoming the perennial issue of consent form length and comprehension.^39, 40, 41^

### Improves scientific literacy

By implementing an interface that participants can access in their own time, there is an additional opportunity for understanding the information provided to patients and participants and reflection on its meaning for their particular circumstances. Initial recruitment will still involve the provision of the information mandated by ethical review, but participants will be able to access additional information if, as and when they wish, from brief lay summaries to peer-reviewed scientific papers. The interface empowers the individual, rather than researchers, to determine how much, and what sort of, additional information they access at any point in time. This is likely to lead to a more realistic understanding of research as an iterative, long-term process; enhance participant confidence in its transparency and accountability; and help to develop more appropriate expectations of what a single piece of research can and cannot achieve. It can enhance scientific literacy and understanding of the societal benefit of medical research.

### Improves transparency and risk management

Finally, dynamic consent provides greater transparency and accountability in the research process as patient samples and data can be tracked across research studies to remove bias and erroneous identification. It provides operational control for managing risk throughout the lifetime of the data holding, providing both an audit process and early warning system of potential security breaches. It also provides the means to contact participants for their opinions on controversial issues and in doing so safeguards public trust and ensures accountability.[Boxed-text bx4]

## CHALLENGES OF IMPLEMENTATION

Implementing a dynamic consent model requires cultural change for both health-care professionals and individuals. It requires partnerships for health that are open, transparent and engaging, and which understand and value the central role that patients have in research as the providers of information and biological material. It also requires development of new policies, standards and ways of working that can accompany this approach. The system must have the technical capacity to interface with the systems of the biobanks and research organisations so it can provide information and feedback to participants. New processes and technology for testing and monitoring the integrity of dynamic consent technologies must be developed, to provide operational control for managing risk throughout the lifetime of the data holding. This requires investment of resources such as time, money, expertise and, most importantly, a commitment to such a vision by clinicians and researchers, health-care services, research institutions and governments. Although there are a number of challenges for implementing dynamic consent, the opportunities that the model will provide, using technology to streamline recruitment, consent and re-consent processes and involve more people in research, will be substantial.[Boxed-text bx5]

## CONCLUSIONS

New technologies have improved biomedical research and have led to significant changes in practice. Dynamic consent is an example of using IT to meet the ethical and legal requirements for consent and also providing a means to communicate, engage and recruit individuals into research.

The initial work for dynamic consent was carried out in the *EnCoRe* project (June 2008 to April 2012).^42^ It was developed in the context of three biobanks – the Oxford Radcliffe Biobank, the Oxford Musculoskeletal Biobank, and the Oxford Biobank. Another high-profile example of the dynamic consent concept is Reg4All.^43^

Currently, dynamic consent systems are being designed for the biobanking context. The technical design of the dynamic consent architecture enables the same interface to be used in clinical or other research contexts. Consent could be obtained for research uses of surplus tissue, *de novo* research projects, organ donation and clinical trials. These consents could support the flow of new knowledge between the laboratory and the clinic that is central to translational research and personalised medicine. Research continues to develop, and new questions arise. As discussion continues about feedback relating to incidental findings discovered during medical research (particularly genomic analysis), the interface would provide a useful means to broach this issue with participants. It could enable researchers to gain a better sense of participants' views on incidental findings and could ultimately provide the opportunity for participants to be better informed and to set specific preferences relating to what feedback they would like, and when and how it is received. Therefore, while dynamic consent is currently a biobanking project, it is also an approach or concept that is being applied more broadly, not only within health but also in other fields.

## Box 5 Challenges of Implementation

The implementation of dynamic consent requires:

- A change in culture for both health professionals and individuals.
- The development of new kinds of partnerships between researchers and patients.
- New policies, standards and ways of working that recognise the valuable role of patients.
- New technologies and processes to provide operational control.
- Time, money, expertise and, most importantly, commitment from clinicians, researchers, health-care services, research and governments.
